# Correction: Chemical stability and interactions in a new antihypertensive mixture containing indapamide and dihydralazine using FT-IR, HPLC and LC-MS methods

**DOI:** 10.1039/c8ra90083c

**Published:** 2018-11-09

**Authors:** Anna Gumieniczek, Justyna Galeza, Anna Berecka, Tomasz Mroczek, Krzysztof Wojtanowski, Katarzyna Lipska, Joanna Skarbek

**Affiliations:** Department of Medicinal Chemistry, Medical University of Lublin Jaczewskiego 4 20-090 Lublin Poland anna.gumieniczek@umlub.pl; Department of Pharmacognosy with Medicinal Plant Unit, Medical University of Lublin Chodźki 1 20-093 Lublin Poland

## Abstract

Correction for ‘Chemical stability and interactions in a new antihypertensive mixture containing indapamide and dihydralazine using FT-IR, HPLC and LC-MS methods’ by Anna Gumieniczek *et al.*, *RSC Adv.*, 2018, **8**, 36076–36089.

The authors regret that incorrect details were given for ref. 19 in the original article. The correct version of ref. 19 is shown in the references section below.

The Royal Society of Chemistry apologises for these errors and any consequent inconvenience to authors and readers.

## Supplementary Material
